# The role of public policy in debt level choices among small-scale manufacturing enterprises in Ethiopia: conditional mixed process approach

**DOI:** 10.1016/j.heliyon.2021.e08548

**Published:** 2021-12-07

**Authors:** Wondemhunegn Ezezew Melesse, Ermias Berihun, Fentahun Baylie, Derbew Kenubih

**Affiliations:** a School of Economics, University of Gondar, Gondar, Ethiopia; b Department of Accounting, University of Gondar, Gondar, Ethiopia

**Keywords:** Debt financing, Small-scale enterprises, Ordered probit, Conditional mixed process, Ethiopia

## Abstract

This paper explores the determinants of debt financing choices among small-scale manufacturing enterprises in Ethiopia—with special focus on the role of government policies. The study exploits survey data gathered from 1321 enterprises in the Amhara region of Ethiopia and employs conditional mixed process (CMP) system estimation technique to test the effect of public policy on firm debt levels. The relevant econometric findings confirm that policy activism through the provision of training and related intervention schemes boosts debt utilization in startup finance mix while it lowers the probability of firms' falling into higher debt levels over time. The results also show that enterprises that had some debt mix in their startup capital are more likely to be in higher debt categories than those enterprises that kick start exclusively with their own internal resources. In addition, the findings also reveal that self-reported profitability, firm age, and ownership structure have strong effects on the degree of firms’ indebtedness. One major bottleneck to the survival and growth of SMEs is their relatively large default rates. One strand of the existing literature shows that firm default rates are strongly correlated with debt levels. As default rates driven by high debt levels have devastating implications for creditors, debtors, and regulators, it is very important to understand the determinants of debt levels. This study is the first to apply conditional mixed process system estimation on firm level data from Ethiopia to test the effects of government policies on debt level choices.

## 1 Introduction

The contribution of small-scale enterprises to gross domestic product (GDP) is higher than that of big companies and small and medium firms constitute the majority of business establishments in developing economies (Bas et al., 2009; Ayyagari et al., 2011). Micro, small, and medium enterprises tend to dominate the global business landscape accounting for more than 95% of the enterprises that generate over 60% of the global private sector employment. Thus, micro firms as well as small and medium enterprises (SMEs) provide badly needed jobs in emerging and developing economies plagued by large and persistent unemployment problems. Moreover, SMEs are usually more labour intensive and make substantial contribution to job creation (Edinburg Group, 2013).

Over the past couple of decades, the government of Ethiopia has given considerable attention to small-scale enterprises in general and to manufacturing firms in particular as an important instrument of poverty reduction and job creation in urban areas. For instance, the Central Statistical Agency of Ethiopia (CSA, 2015) reported that as of 2013/2014 there were over 116 thousand small-scale manufacturing enterprises in Ethiopia, employing more than 1.7 million workers and contributing about 10.88 billion Birr (approximately $241 million at current exchange rate) worth of GDP. Moreover, in the second Growth and Transformation Plan (Ministry of Finance and Economic Development of Ethiopia, 2010) the government of Ethiopia envisaged the GDP contribution of small and micro manufacturing establishments to rise from 1.1% in 2014/15 to 2% by 2019/20. The attention given to the small-scale manufacturing sector has only intensified under the new administration. The new ten year economic development plan for 2021–2030 aims at creating 2 million micro enterprises of which 10% to be promoted to small-scale and 1% to medium-scale manufacturing enterprises in order to enhance their contribution to employment and industrial development over a ten year period (Planning and Development Commission of Ethiopia, 2020).

Despite their economic and employment significance, small-scale firms face a number of obstacles, especially in low-income countries. The most important impediment concerns the limited existence of responsive financial system. Ethiopia exhibits one of the most underdeveloped financial systems in the world. For instance, the 2019 Global Competitiveness Report (GCR) indicated that Ethiopia's overall competitiveness rank was 126 out of 141 countries. In addition, the report's 9^th^ pillar components that capture financial system development show a rank of 103/141 for domestic credit to private sector as percentage of GDP; 114/141 for access to SME financing and 124/141 for soundness of banks (World Economic Forum, 2019). Ethiopia lags far behind other African countries in terms of access to formal financial institutions. Preference for informal saving clubs, pervasive unemployment, low income, distance, high cost of borrowing, and documentation barriers are among the most prominent bottlenecks to financial inclusion in Ethiopia (Tekeste and Azadi, 2020).

Currently, the financial system in Ethiopia is dominated by banks, insurance companies and microfinance institutions without functioning stock markets. The banking sector is characterized by dormant^1^ interbank transactions and excess liquidity which suggest for the presence of excess capacity within the financial system. Despite the dominance of Ethiopia's financial system by banks, the government's bold financial inclusion measures have encouraged the development of microfinance institutions and savings and credit cooperative unions, which have been instrumental in expanding financial services to previously unbanked segments of the economy (International Monetary Fund, 2019).

Another important bottleneck to the survival and growth of SMEs is their relatively large default rates. While there is mounting evidence about the positive effects of debt financing on several metrics of firm growth (see, for instance, Levine, 2004; Cornille et al., 2019 and Gó mez, 2019), widespread default and delinquency can be destabilizing and costly. One strand of the existing literature shows that firm default rates are strongly correlated with debt levels (Fidrmuc and Hainz, 2010; Abu et al., 2017; Osman and Ramakrishna, 2017). In Ethiopia, studies conducted in different parts of the country have shown that default and delinquency rates are especially very high among small and micro borrowers where default rates reach as high as 50 percent (e.g. Assfaw et al., 2015; Firafis, 2015; Osman and Ramakrishna, 2017; Girma, 2018). As default rates driven by high debt levels have devastating implications for creditors, debtors, and regulators, it is very important to understand the determinants of debt levels.

This paper, therefore, focuses on small scale manufacturing enterprises in a low-income economy to isolate the determinants of different levels of debt with special focus on the role of government policy interventions. The contribution of this paper to the existing literature rests on testing whether policy and regulatory environments affect the nature of financing mix used by firms (Bortolotti et al., 2011; Faulkender and Petersen, 2006). To the best of our knowledge, this study is the first in the context of Ethiopia to test the effects of government policies on patterns of firm financing behaviour using data from small-scale manufacturing establishments. As described earlier small-scale manufacturing firms are important sources of employment and income and they warrant investigation on their own because of their potential to transform the largely agrarian economy of Ethiopia to one dominated by industry and service sectors. While there are very few studies on determinants of leverage in Ethiopia (e.g. Umer, 2014), another contribution of our study to the existing body of knowledge is that we employ conditional mixed process approach that accounts for possible endogenous feedback effects. In addition, the results of the study will be of paramount importance to concerned policy practitioners who could be interested to gauge whether the various bureaucratic interventions at federal and local levels have any association with firm debt levels.

The relevant econometric findings confirm that policy activism through the provision of training and related intervention schemes lower the probability of firms' falling into higher debt levels. The results also show that enterprises that had some debt mix in their startup capital are more likely to be in higher debt categories than those enterprises that kick start exclusively with their own internal resources. In addition, the findings also reveal that self-reported profitability, firm age, and ownership structure have strong effects on the degree of firms’ indebtedness.

The remainder of the paper is organized as follows: Section 2 discusses the relevant theoretical and empirical literature. Overview of data and the methodology is presented in section 3. Section 4 bears the discussion of empirical results, and finally, section 5 offers conclusion and recommendations.

## 2 Literature review

### 2.1 Theoretical review

Modigliani and Miller (1958) provided the launching pad for the theory of capital structure. They proved that without corporate taxes, bankruptcy risks, and related distortions, a firm's balance sheet structure would have no effect on its market value if capital markets are competitive. Overtime, several scholars have offered alternative theoretical frameworks that mainly helped rectify many of the conceptual loopholes in the Modigliani-Miller model, which, in its original form, had limited empirical relevance.

One such alternative framework is the trade-off (TOT) theory that suggests that financing options are haunted by the need to ponder the relative costs and benefits of utilizing external financial resources. The model views a firm's choice of optimal debt-equity mix as a function of three parameters: taxation, liquidation risks, and conflicts among key agents. If the firm decides to use debt, for instance, it enjoys reduction in its corporate tax obligations and boosts the after-tax income at its disposal. In consequence, tax advantage and the market value of the firm co-move in the same direction as a result of firms' efforts to align the tax advantages of more debt with increasing probability of bankruptcy risks (Kraus and Litzenberger, 1973; Myers, 2001).

Jensen and Meckling (1976) provided another refinement where financing mix is regulated by agency costs stemming from conflicts of interest that involve managers, shareholders, and creditors/bondholders. For instance, in Harris and Raviv (1990) though shareholders and/or creditors want to liquidate the firm, managers would rather struggle to extend the life of the firm in question to protect their own economic advantage. In addition, Stulz (1990) also suggests managers have vested interests to invest funds at their disposal despite shareholders’ preferences for cash dividend receipts.

The third major framework in the capital structure literature is the pecking order theory (POT) that was introduced by Donaldson (1961) and was later formalized by Myers and Majluf (1984). The POT of Myers (1984) and Myers and Majluf (1984) did not look for optimal leverage ratio; instead it can be viewed as an alternative model to the TOT. This particular model offers explanations as to why successful business establishments rely a lot more on internal resources and financially less successful firms resort to debt financing as their internal finances are meager. Even though the POT was developed to analyze the financing structure of large corporations, its relevance to the study of small-scale-enterprise financing cannot be discounted (Mlohaolas et al., 1998; Osei-Assibey et al., 2012). For one thing SMEs are opaque and entail considerable information-related costs (Psillaki, 1995). Moreover, SME owners want independence and prefer to maintain control over their own firms (Berggren et al., 2000) and SMEs are likely to be infested with adverse selection and moral hazard problems (Frank and Goyal, 2003).

Graham and Leary (2011) reviewed several attempts aimed at overcoming the empirical shortcomings of the traditional capital structure theories. The authors emphasized efforts that considered, among others, mis-measurement of key variables in empirical studies, the effect of leverage on nonfinancial stakeholders, supply side effects on business capital structure, the role of financial contracts, bias in estimates of leverage adjustment speeds, and capital structure dynamics. Related developments point out that firms' leverage structure can be influenced by several other factors that include source of capital (Faulkender and Peterson, 2006), macroeconomic and institutional factors (Kenourgios et al., 2019), unobserved firm fixed effects (Nguyen et al., 2019), credit supply shocks or systemic financial meltdown (D'Amato, 2019; Demirgüç-Kunt et al., 2020). Other scholars also argue that owner-manager specific characteristics--such as education and gender--play considerable roles in shaping the financing preferences and leverage structures of business establishments (Abor, 2008; Nawi, 2015).

### 2.2 Empirical review

Empirically numerous studies have examined the sources, patterns and implications of different financing preferences of firms. Concentrating on Brazilian small and medium firms, Forte et al. (2013) found negative association between profitability and leverage and positive correlation between asset growth and leverage. Bulent et al. (2013) investigated the determinants of capital structure among unbalanced panel of 11,726 firms in Turkey observed between 1996 and 2009. In their analysis firm size was found to be the most significant firm attribute than firm age or industrial membership in predicting financing structure. Looking at another emerging economy, ur Rehman et al. (2017) examined 760 firms in China exploiting extensive dataset stretching from 2001 to 2013. The main results indicated that Chinese firms have target levels of leverage and that they adjust towards those preferred levels.

In the context of developing countries, Salawu and Agboola (2008) exploiting data from Nigeria found that profit, tangibility and firm size have positive correlation with total debt and long-term debt while growth opportunities had negative relationship with total debt. Gwatidzo et al. (2016) used quantile regression for South Africa and confirmed that the effects of key standard predictors do not vary with different levels of leverage. Umer (2014) explored the determinants of capital structure among a panel of 37 large taxpayer firms in Ethiopia. The author found positive correlation between leverage and firm size, firm age, tangibility, liquidity position and non-debt tax advantages while profit, income volatility and dividend payments had negative co-movement with degree of firm indebtedness. Focusing on small and micro firms in rural Ghana, Osei-Assibey et al. (2012) studied the determinants of financing patterns by exploring a wide range of financing options. Their findings indicate that younger firms tend to prefer low-cost, less-risky or less-formal external sources starting from internal finances.

Beyond the standard determinants, some scholars have accentuated the importance of owner-manager specific characteristics. For instance, in a study of small and medium enterprises in Malaysia, Nawi (2015) found that owner's networking and social relationship had positive influence on the probability of debt financing while owner age and education had little effect on firms' balance sheet structures. In addition, the study documented that ethnic minorities were more likely to rely on funds from families and friends and less likely to secure external loans suggesting the possibility of discrimination in the credit market against minority borrowers. Other studies that account for owner-specific features in the capital structure literature include Kuruppu and Azeez (2016) for Sri Lanka as well as Abor (2008) and Osei-Assibey et al. (2012) for Ghana.

Still very important, the policy environment in which firms operate can also shape their financing choices. For instance, it is now widely recognized that tax policy considerations and probability of bankruptcy risks influence firm financing choices (Kraus and Litzenberger, 1973; Myers, 2001). Overesch and Voeller (2010) confirmed that high tax benefit of debt has significant positive effect on a firm's financial leverage. In addition, they found that the leverage structures of smaller firms adjust more readily to changes in the tax benefit of debt. Zhang et al. (2015) have shown that leverage ratio declines with increasing economic policy uncertainty. Moreover, Cao et al. (2013) found that highly indebted firms tend to reduce their leverage levels while less indebted firms are inclined to delay borrowing in times of high political uncertainty. Bortolotti et al. (2011) found higher leverage among firms that are privately owned and subject to regulation by an independent regulatory authority. Similarly, Faulkender and Petersen (2006) identified significantly more leverage among firms with exposure to debt rating and having access to the public bond markets.

In Ethiopia, federal and local governments provide several financial and non-financial support packages as part of an effort to encourage small-scale enterprise development as a tool for poverty reduction and job creation. These packages include training, technology transfer, and related business development services (BDS) as well as organizational support to the youth by the government during business formation process (Federal Democratic Republic of Ethiopia, 2011). These government-provided services are used as proxy for policy activism. The expectation is that firms that have access to such services and facilities could develop leverage structures that are significantly different from those enterprises that do not enjoy similar entrepreneurial support facilities.

In light of the above discussion, the major hypotheses the study aims to test are the following:

H1 Access to business development services (BDS) has no effect on debt financing level.

H2 Government support in group business formation process has no effect on debt financing level.

In addition to the inconclusive nature of the existing evidence on the debt-policy nexus, there are very few studies on determinants of leverage in low-income economies in general and in Ethiopia in particular. The only study we are aware of is Umer (2014) who investigated determinants of leverage among large scale taxpayer companies based in Addis Ababa. We intend to fill three important gaps. The first is our focus on small-scale manufacturing enterprises which the government recognizes as critical instruments to achieve economic transformation and poverty reduction. Since so much research in Ethiopia is concentrated on service and agriculture, we believe due consideration needs to be given to the manufacturing sector going forward. In that sense we hope our study will be a stepping stone for further studies on debt-policy nexus in the manufacturing industry. Secondly, to the best of our knowledge, this study is the first attempt on analyzing the possible connection between policy activism and debt level choices. The final contribution of our paper is to take the possibility of endogenous feedback relationships which if ignored biases the estimation results. We employ conditional mixed process estimation strategy proposed by Roodman (2011) to take care of possible endogenous feedback effects from debt level to profit and startup capital mix and vice versa.

## 3 Materials and methods

### 3.1 Data source

The data used in this paper is part of a broader dataset collected in 2016/17 fiscal year as part of a mega research project to assess the status of small and micro manufacturing enterprises in the Amhara region of Ethiopia. Available information was collected by administering structured questionnaires to focal persons (including owners and managers) in 1381 small and micro manufacturing enterprises. These enterprises were scattered across eleven zonal capital towns of the same region. Ultimately 1321 enterprises filled in and returned the distributed questionnaires giving rise to a response rate of about 96 percent. The available information was gathered from the owners/managers of the target firms by employing multi-stage stratified simple random sampling techniques. In the first stage, the Amhara region was selected as this region housed the university that supported the specific research as part of an effort to understand the socio-economic problems and contribute to the local community development needs. Inclusion of other regions in the country was not possible because of financial constraints. In the second stage, the team leaders decided to cover all the eleven zonal capital towns of Amhara region as the vast majority of the small and micro manufacturing enterprises was concentrated in those towns. In each town, the enumerators contacted the concerned technical, vocational, and enterprise development office personnel for lists of the target firms. In the final stage, the questionnaires were distributed to proportionally allocated and randomly selected enterprises to make sure that each sub-sector receives fair representation in the total sample.

### 3.2 Econometric model

Drawing on Wooldridge (2002), let y be an ordered-response variable taking on the values of 0, 1, 2,. . ., J. For some known integer J the relevant ordered probit model for y (conditional on explanatory variables x) can be derived from a latent variable model. Denoting this latent variable by y the relevant relationship can be determined by:

(1) $$y_{1}=y_{2}\varphi +xb_{1}+e_{1},\;e_{1}|x,y_{2}∼N\left(0,1\right)$$

where b is a k-by-1 parameter vector to be estimated and x is a vector of k predictors that potentially affect the dependant variable and e is the model residual term. In this study $y_{1}$ equals 0 if the specific firm reported no debt at all; 1 if it has a debt ratio level above 0 but below 50 percent; and 2 if the debt ratio level at the time of the survey exceeds 50 percent. Thus, J = 0, 1, 2 represent absence of any debt, moderate level of leverage and significantly higher level of debt to asset ratio, respectively, in the sampled small and micro manufacturing firms included in the present analysis.

Many previous studies (e.g. Beck et al., 2004; Ossei-Assibey et al., 2012; Brunzell et al., 2015; and Belas et al., 2018) studied firm financing structures under the implicit assumption that all right-hand-side variables are exogenous. This implicit assumption ignores the possibility that causality could run in both directions. For instance, the nature of startup financing mix has implications for current leverage structure. It is also possible to argue that current financing structure could influence the nature of startup financing in existing and newly created enterprises. Ignoring such potential spillover effects running in both directions could constitute a major model misspecification if they are present. A similar argument can be made in view of the profitability variable.

(2) $$y_{2}=z\rho +xb_{2}+e_{2},\;e_{2}|x,z∼N\left(0,1\right)$$

In Eq. (2) above, $y_{2}$ stands either for startup capital mix (which is binary) or profitability changes (categorical variable with three levels—decreased, unchanged or increased). The variable z captures the perception of respondents about the importance of rental costs of production and/or sales premises. This variable is used as an excluded instrument for $y_{2}$ and is expected to have strong correlation with startup financing mix and profitability level. In particular, ρ is expected to be negative as perceptions about the importance of rental costs of sales and production premises should correlate inversely with both profitability and the use of debt in startup finance.

Roodman (2011) has developed fully observed recursive conditional mixed process (CMP) framework that extends the standard seemingly unrelated regression (SUR) model into a non-linear setting. The CMP approach facilitates inter-dependent equations (as in Eqs. (1) and (2) above) to be correlated across a recursive system where the dependent variables could be binary, ordered, or truncated. The CMP framework allows the endogenous independent variable in one equation to act as a dependent variable in another equation through recursive arrangement. The system CMP model is superior to the standard single equation estimation techniques as long as the correlation coefficient of the error terms between the two equations is statistically significant.

### 3.3 Description of variables

#### 3.3.1 Dependent variable

Several studies have used level categories to study degree of indebtedness among individuals, households, and firms (e.g. Zhao et al., 2006). In this study, the dependant variable encompasses three alternatives based on the estimated debt to asset ratio reported by the sampled respondents. The lowest category (no debt) has been coded as 0; the middle category for debt ratio between 0 and 50 percent coded as 1; and the uppermost category representing debt-to-asset ratio in excess of 50 percent coded as 2. These ratios were constructed based on respondents’ estimation of their assets and debt levels. As obtaining exact figures were difficult due to absence of recording, the respondents were asked to choose among the specified leverage categories. The three category choices may appear arbitrary; however, given the practical limitations, this approach minimizes classification errors. The respondents can easily memorize whether their debt-to-asset ratio is below or above the 50% threshold. However, they could find it hard to distinguish whether their leverage is above or below, say, the 75% threshold. Some authors (e.g. Zhao et al., 2006) have considered levels with four categories where the uppermost group includes those consumers with indebtedness exceeding 20%. Such refinement was not possible in this study due to absence of financial recordings at the target enterprises.

#### 3.3.2 Independent and control variables

This study incorporates numerous independent variables drawing from the existing literature. In order to capture potential effects of government policy, the study includes two proxy variables, namely, access to government-provided training, technology transfer and related business development services (BDS) and existence of local government initiative in organizing job seekers to form groups and start business. Finally, the study includes other standard control variables that affect current degree of firms’ indebtedness (see Table 1 for variable definition and measurement).

**Table 1:** Definition, sources, and expected signs of model variables.

Variable	Definition	Sources	Expected sign
Firm age	In categories of years. Those firms younger than three years being the benchmark.	Klapper et al. (2006); Umer (2014)	+/-
Firm size	Total number of employees	Antoniou et al. (2008); Salawu and Agboola (2008); Osei-Assibey et al. (2012); Bulent et al. (2013); ur Rehman et al. (2017);	+/-
Ownership type	Solo = 1, group = 0	Klapper et al. (2006); Osei-Assibey et al. (2012); ur Rehman et al. (2017)	+/-
Profit over time	Decreased (benchmark), unchanged, increased	Antoniou et al. (2008); Salawu and Agboola (2008); Bulent et al. (2013); Umer (2014); Gwatidzo et al., 2016)	+/-
Access to training and other business development services (BDS)	Access = 1, no access = 0	Osei-Assibey et al. (2012); Kuruppu and Azeez (2016)	+
Owner education	Basic (benchmark), primary, secondary, college+	Scherr et al. (1993); Abor (2008); Osei-Assibey et al. (2012); Nawi (2015); Kuruppu and Azeez (2016)	+
Owner sex	Male = 1, female = 0	Scherr et al. (1993); Abor (2008); Osei-Assibey et al. (2012); Kuruppu and Azeez (2016)	+/-
Location	Current firm location	Abor (2008); Osei-Assibey et al. (2012)	+/-
Sub-sector	Major marketed product	Klapper et al. (2006); Kuruppu and Azeez (2016)	+/-
Startup capital mix	Some or all loan = 1, no loan = 0		+
Government support in firm creation	Present = 1, absent = 0	Bortolotti et al., (2011); Faulkender and Petersen (2006)	**+**

## 4 Results and discussion

### 4.1 Descriptive results

Descriptive statistics of the major model variables are presented in Table 2. Most of the enterprises in the sample are young as the average age of the business establishment is about four years with standard deviation of 3.45. Similarly, the typical enterprise had an employment level of about five workers which suggests that the distribution is tilted towards micro-type^2^ businesses. Responding to business environment perception, most (about 60%) said profitability of their business increased since they started operation; about 27% of the sample respondents saw no discernible change, and the remaining 13% experienced shrinkage. When asked about the mix of the financing scheme when they started operation, about 64% reported that they relied exclusively on their own internal resources and the remaining fraction used some mix of debt and own finances.

**Table 2:** Descriptive statistics of variables.

Variable	Min	Max	Mean	St.dev
a) Continuous				
Enterprise age	0.2	45	4.25	3.45
Employees	1	30	5.13	4.54

b) Categorical		
Sex of owner	Percentage	Counts
Male	70.6	932
Female	29.4	389
Education of owner		232
College+	17.6	613
Secondary	46.4	312
Primary	23.6	164
Basic	12.4	
Profit since establishment		
Increased	60.4	798
Unchanged	26.8	354
Decreased	12.8	169
Mix of startup capital		
Own funds	64.3	849
Full or partial loan	35.7	472
Current access to business development services		
Yes	69	908
No	31	393
Enterprise formation process		
By government initiative	14.3	189
Other	85.7	1,132
Ownership type		
Individual	52.6	695
Group/family	47.4	626
No. of observations		1321

Source: Author's own computation based on survey data.

Table 2 also shows that about 69% of the enterprises had access to some type of government-provided business development services that include training, technology extension, work place management and other related skill transfers. Most enterprises (about 86%) joined the manufacturing sector on their own initiative and the remaining 14% through government encouragement and support. In the econometric part, we will formally test whether these two variables influence leverage positions of firms in a systematic way. The incorporation of these two qualitative variables in the variety of regressions performed in Tables 4 and 5 and 7 amounts to a test of the role of public policy in shaping firms’ debt financing patterns.

Stylized facts on the sampled firms’ financing profiles^3^ are provided in Table 3 under panels (a) and (b). The results summarize two-way-cross tabulations of recent-credit-access history and leverage positions with the structure of startup finance. The numbers in brackets indicate cell fractions relative to row totals. For instance, among 254 enterprises that started production relying on exclusively borrowed funds, an overwhelming majority (73%) had recent access to credit and about 68% had some amount of debt on their balance sheet. By contrast, out of the 849 firms without any debt upon establishment, only 191 (22%) were able to access external finance and only 24% had non-zero leverage positions at the time of the interview. These descriptive results suggest that there is considerable *debt inertia* in the sense that those enterprises that get into business with some liability are more likely to have access to credit market and to maintain a certain degree of indebtedness over time.

**Table 3:** Financial profile of sample firms.

a)Link between startup finance and recent access to credit			
Startup finance	Recent access to credit		Total
	No	Yes	
Loan only	69 (27%)	185 (73%)	254
Own funds only	658 (78%)	191 (22%)	849
Some mix	72 (33%)	146 (77%)	218
Total	799	522	1,321

b) Link between startup finance and current leverage status				
Startup finance	Current leverage status			Total
	0%	<50%	≥50%	
Loan only	81 (32%)	136 (53.5%)	37 (14.5%)	254
Own funds only	647 (76%)	150 (18%)	52 (6%)	849
Some mix	68 (31%)	119 (55%)	31 (14%)	218
Total	796	405	120	1,321

Source: Author's own computation based on survey data.

### 4.2 Econometric results

#### 4.2.1 Ordered-probit estimation

Table 4 presents the corresponding marginal effects from ordered-probit model for the fully-fledged specification in the baseline^4^ setup. The results show that using some debt in startup finance lowers the probability of being in no-debt category by about 38% which is significant at 99 percent confidence level. Similarly, using some debt in startup finance raises the probability of being in moderate and high leverage categories by about 25% and 13%, respectively. The current finding is in congruence with the descriptive results discussed earlier on the presence of debt inertia. Other studies have also established the existence of persistence in leverage structure by employing a diversity of materials and statistical techniques (see, for instance, Antoniou et al., 2008 and ur Rehman et al., 2017).

**Table 4:** Ordered-probit marginal effects: baseline model.

Dependent variable: debt level categories			
Predictors	Pr.(no debt)	Pr. (lev. < 50%)	Pr. (lev. ≥50%)
Startup finance (debt = 1)	-0.375∗∗∗ (-13.74)	0.250∗∗∗ (11.28)	0.125∗∗∗ (10.59)
Firm age ≥ 4 & ≤6 years	-0.010∗∗ (-0.35)	0.007 (0.35)	0.004 (0.35)
Firm age >6 & ≤ 8 years	0.116∗∗ (2.50)	-0.082∗∗ (-2.36)	-0.034∗∗ (-2.78)
Firm age >8 years	0.115∗∗ (2.39)	-0.081∗∗ (-2.26)	-0.034∗∗ (-2.66)
Firm size (total workforce)	-0.006∗∗ (-2.10)	0.004∗∗ (2.08)	0.002∗∗ (2.08)
Profitability -unchanged	0.090∗∗ (2.00)	-0.053∗∗ (-2.06)	-0.037∗ (-1.88)
Profitability -increased	0.144∗∗∗ (3.52)	-0.089∗∗∗ (-3.81)	-0.054∗∗ (-2.98)
Ownership (solo = 1)	0.021 (0.71)	-0.014 (-0.71)	-0.006 (-0.71)
Firm formation (government = 1)	-0.025 (-0.65)	0.0170 (0.65)	0.008 (0.67)
Access to BDS (yes = 1)	0.032 (1.14)	-0.022 (-1.14)	-0.011 (-1.14)
Education –primary	-0.019 (-0.40)	0.013 (0.40)	0.006 (0.41)
Education - secondary	-0.036 (-0.84)	0.024 (0.83)	0.011 (0.87)
Education – college+	-0.090∗ (-1.85)	0.059∗ (1.82)	0.031∗ (1.86)
Sex (male = 1)	-0.025 (-0.87)	0.017 (0.87)	0.008 (0.87)
Observations	1,321		

Note: ∗∗∗<0.01, ∗∗<0.05, ∗<0.1.

Firm age (with those below three years as a benchmark) has non-linear effect on leverage positions of target firms. For instance, those firms older than 8 years have about 12% additional probability of being in no-debt category while the corresponding probabilities for being in moderate and high levels of leverage decline by about 8% and 3.4%, respectively. The marginal effects are significant at 95 percent confidence level. These findings indicate that maturity has inverse relationship with the likelihood of using higher levels of debt in clear contrast with the prediction of the POT. The present finding is inconsistent with the findings of Abor (2008); Osei-Assibey et al. (2012), and Umer (2014), for example, who find positive relationship between firm age and leverage. It is, however, in line with the results of Gwatidzo et al. (2015) that showed that for some quantile of the data distribution, older and more established firms tend to be less leveraged. The complete set of marginal effects in Table 4 confirms that improvement in profitability across time has strong negative correlation with leverage level positions. For example, those enterprises that reported increased profitability saw a 14% increase in the probability of being in no-debt category while the corresponding probabilities of staying in moderate and high levels of leverage declined by about 9% and 5%, respectively. Consistent with the pecking order theory, these findings imply more profitable enterprises tend to rely on internal financing schemes. Negative correlations (with and without qualifications) between leverage and different profit measures have been confirmed by previous studies that include, among others, Klapper et al. (2006), Abor (2008), Bas et al. (2009), Forte et al. (2013), and ur Rehman et al. (2017). The present finding is, however, inconsistent with Scherr et al. (1993) who confirmed the existence of significant positive correlation between business income and the level of firm debt.

The marginal effects in Table 4, also reveal that entrepreneurs with at least college education attainment see increased probabilities of exposure to higher level of debt financing compared with the literate (the base group)—results which are significant at 90 percent confidence level. This finding clearly contradicts the results of Nawi (2015) who found no significant association between entrepreneurs’ education and related financial decisions of SMEs. Other studies by Abor (2008), Osei-Assibey et al. (2012) and Kuruppu and Azeez (2016) have found some subtle effects of education on the financing structure of firms.

#### 4.2.2 Conditional mixed process (CMP) system estimation

Table 5 displays the marginal effects for both the leverage and startup financing equations. The degree of correlation between the two equations (Atanhrho) is -1.537 and is statistically significant at 99 percent confidence level—which justifies the use of system estimation confirming the cross-equation interdependence in both directions. However, the possibility of endogenous feedback from the profitability equation is not supported empirically as the estimated correlation measure is statistically insignificant (see the coefficient estimates included in appendix table 7). In both cases, a new categorical variable called ‘rental cost’ was used as a possible excluded instrument. The rental cost variable is a binary indicator that equals 1 if the specific firm reported that rental cost of production and/or sales premises is important and zero otherwise.

**Table 5:** Determinants of debt levels: marginal effects from CMP system estimation for endogenous startup finance mix.

	Debt level equation			Startup finance equation
	Pr. (no debt)	Pr.(lev. <50%)	Pr.(lev. ≥50%)	Pr.(debt = 1)
Startup finance (debt = 1)	-0.508∗∗∗ (-77.89)	0.108∗∗∗ (13.12)	0.401∗∗∗ (44.30)	
Firm age ≥ 4 & ≤ 6 years	0.006 (0.38)	-0.001 (-0.38)	-0.048 (-0.38)	0.010 (0.13)
Firm age >6 & ≤ 8 years	0.051∗ (1.82)	-0.011∗ (-1.77)	-0.041∗ (-1.83)	-0.022 (-0.17)
Firm age >8 years	0.063∗∗ (2.21)	-0.013∗∗ (-2.15)	-0.050∗∗ (-2.22)	0.081 (0..63)
Firm size (total labour force)	-0.001 (-0.66)	0.000 (0.65)	0.001 (0.66)	0.015∗ (1.80)
Profitability-unchanged	0.039 (1.56)	-0.008 (-1.54)	-0.031 (-1.56)	0.089 (0.73)
Profitability –increased	0.069∗∗ (3.09)	-0.015∗∗ (-2.92)	-0.055∗∗ (-3.12)	0.081 (0.73)
Ownership (solo = 1)	0.034∗∗ (2.10)	-0.008∗ (-1.73)	-0.027∗∗ (-2.09)	0.137∗ (1.75)
Firm formation (government = 1)	0.038∗ (1.75)	-0.008∗ (-1.76)	-0.030∗ (-1.75)	0.367∗∗∗ (3.39)
Access to BDS (yes = 1)	0.028∗ (1.79)	-0.006∗ (-1.76)	-0.022∗ (-1.79)	0.136∗ (1.79)
Education-primary	-0.033 (-1.29)	0.007 (1.28)	0.026 (1.29)	-0.072 (-0.59)
Education-secondary	-0.013 (-0.53)	0.003 (0.53)	0.010 (0.53)	0.053 (-0.59)
Education-college+	0.015 (0.55)	-0.003 (-0.55)	-0.012 (-0.55)	0.374∗∗ (2.79)
Sex (male = 1)	-0.014 (-0.86)	0.003 (0.86)	0.011 (0.86)	-0.038 (-0.50)
Rental cost				-0.147∗∗ (-2.50)
Observations	1321			1321
Atanhrho	-1.537∗∗∗ (-7.34)			

Note: Z values are provided in brackets ∗∗∗<0.01, ∗∗<0.05, ∗<0.1.

The marginal effects presented in Table 5 suggest that considering the endogeneity of startup finance mix reveals a number of interesting results. First, the policy activism variables become statistically significant at 90 percent confidence levels. The results indicate that firms which have government support during their formation process and firms having exposure to business development services see increased probability of being in the zero-debt and decreased probabilities of being in higher debt categories. Specifically, having government support during firm formation and access to training and related business development services lower the probability of falling into the highest debt category by 3 percent and 2.2 percent, respectively. This could be due to the fact that firms that get bureaucratic support and entrepreneurial training are more successful in their business performance which in turn lowers the odds of being or remaining in higher levels of indebtedness. Thus, these results indicate that using CMP approach gives us significantly different results from those obtained by using single-equation ordered-probit models. These findings are in line with previous studies that revealed significant effects of policy and political conditions on firm debt levels (e.g. Faulkender and Petersen, 2006; Bortolotti et al., 2011; Cao et al., 2013; Zhang et al., 2015).

The results in Table 5 also confirm that policy activism does not reduce the use of debt when firms start their operation. In fact, access to BDS and government support during firm formation process raise the probability of using debt in startup finance by 14% and 37%, respectively, which are significant at conventional confidence levels. Thus we can conclude that consistent with government goals, policy support packages improve debt finance utilization when a firm starts production but lowers the intensity over time. As shown in Table 2 the average age of the sampled enterprises in this study is about 4.25 years. Thus policy support packages help firms reduce their debt levels over time while they do not discourage firms from using debt at the beginning of their operation. One possible explanation for this observation is that as shown in Table 5, profitability (standard measure of business performance) reduces the probability of falling into higher levels of indebtedness. In fact, the main goal of these policy packages is to help improve the growth and development of such enterprises through time (Federal Democratic Republic of Ethiopia, 2011).

Second, the sign and significance of startup financing mix, firm age, and profitability are also preserved in the system estimation results. Third, the ownership variable, which was insignificant so far, now enters significantly at 95 percent confidence level. This specific result confirms that individually owned firms see lower probabilities of being in higher debt levels. The correlation between ownership structure of the firm and financing patterns was also confirmed by previous studies (e.g. Klapper et al., 2006; ur Rehman et al., 2017).

Finally, firm size, ownership structure, government support in business formation process, education attainment, and perception about the role of rental cost are significant drivers of the mix of startup finance. For instance, having college education raises the probability of using debt in startup financing by about 37%, a result significant at 95 percent confidence level. This implies that education affects leverage level indirectly through its effect on the choice of startup finance mix. This contradicts the results of single equation studies that showed that education has direct effect on firm debt levels (Abor, 2008; Osei-Assibey et al., 2012; and Kuruppu and Azeez, 2016).

## 5 Conclusion and recommendations

Understanding the patterns and drivers of firm balance sheet composition is important from macro- and micro-economic points of view. This paper explores the determinants of debt levels among small-scale manufacturing enterprises in Ethiopia. The study relies on survey data gathered from Amhara region during 2016/17 fiscal year and employs descriptive and conditional mixed process (CMP) estimation techniques to isolate the effects of both standard and heterodox factors on firm financing preferences. The relevant econometric findings confirm that policy activism through the provision of training and related intervention schemes lower the probability of firms' falling into higher debt levels. The results also show that enterprises that had some debt mix in their startup capital are more likely to be in higher debt categories than those enterprises that kick start exclusively with their own internal resources. In addition, the findings also reveal that self-reported profitability, firm age, and ownership structure have strong effects on the degree of firms' indebtedness. Since policy activism was found to have significant negative relationship with the likelihood of falling into higher debt levels, concerned policymakers should strengthen existing financial and non-financial policy intervention packages in order to lower the probability of firms’ falling into destabilizing debt levels.

## Declarations

### Author contribution statement

Wondemhunegn Ezezew: Conceived and designed the experiments; Performed the experiments; Analyzed and interpreted the data; Contributed reagents, materials, analysis tools or data.

Ermias Berihun: Conceived and designed the experiments; Performed the experiments; Analyzed and interpreted the data; Contributed reagents, materials, analysis tools or data; Wrote the paper.

Fentahun Baylie and Derbew Kenubeh: Analyzed and interpreted the data; Wrote the paper.

### Funding statement

This research was supported by the 10.13039/501100007861University of Gondar, Gondar, Ethiopia.

### Data availability statement

Data will be made available on request.

### Declaration of interests statement

The authors declare no conflict of interest.

### Additional information

No additional information is available for this paper.

## Figures and Tables

**Table 6:** Contingency correlation test results for dependence among qualitative predictors.

	(a)	(b)	(c)	(d)	(e)	(f)	(g)	(h)	(i)	(j)	(k)
Startup (a)	1										
Firm age (b)	0.020	1									
Profit (c)	0.059	0.067	1								
Credit access(d)	0.468	0.131	0.098	1							
BDS(e)	0.065	0.052	0.059	0.039	1						
Gov't role (f)	0.140	0.075	0.058	0.046	0.019	1					
Ownership (g)	0.150	0.113	0.069	0.045	0.032	0.383	1				
Education (h)	0.109	0.075	0.124	0.112	0.071	0.013	0.062	1			
Sex (i)	0.036	0.063	0.090	0.067	0.029	0.049	0.012	0.123	1		
Location (j)	0.163	0.197	0.209	0.192	0.257	0.166	0.305	0.222	0240	1	
Subsector (k)	0.099	0.136	0.129	0.139	0.103	0.087	0.118	0.127	0.463	0.183	1

Notes: Cramer's V correlation stats are all below 0.5 signaling the absence of serious linear dependence among the target qualitative predictors.

**Table 7:** Determinants of debt levels: coefficients from CMP system estimation for possible endogenous profitability.

Dependent variable: debt level categories		
	Debt level equation	Profitability equation
Firm age ≥ 4 & ≤ 6 years	0.067 (0.92)	-0.079 (-1.12)
Firm age >6 & ≤ 8 years	-0.235∗ (-1.69)	-0.081 (-0.69)
Firm age >8 years	-0.227∗ (-1.78)	0.057 (0.39)
Firm size (total labour force)	0.019∗∗ (2.55)	-0.004 (-0.53)
Profitability-unchanged	1.329 (1.50)	
Profitability –increased	0.473 (0.99)	
Ownership (solo = 1)	0.033 (0.46)	-0.059 (-0.84)
Firm formation (government = 1)	0.158 (1.49)	0.066 (0.68)
Access to BDS (yes = 1)	-0.067 (-0.93)	0.103 (1.50)
Education-primary	-0.046 (-0.40)	0.086 (0.78)
Education-secondary	0.030 (0.26)	0.145 (1.43)
Education-college+	0.284∗∗ (1.98)	0.117 (0.99)
Sex (male = 1)	0.108 (1.35)	-0.181∗∗ (-2.59)
Rental cost		-0.105∗ (-1.69)
Observations	1321	1321
Atanhrho	-0.585 (-1.40))	

Note: Z values are provided in brackets ∗∗∗<0.01, ∗∗<0.05, ∗<0.1.

**Table 8:** Coefficients from CMP system estimation with and without robust standard errors for possible endogenous startup financing mix.

Dependent variable: debt level categories						
	Debt level equation				Startup equation	
	Baseline CMP			CMP with robust SEs	Baseline CMP	CMP with robust SEs
Startup finance (debt = 1)	2.203 (35.20)∗∗∗	2.203∗∗∗ (33.03)				
Firm age ≥ 4 & ≤ 6 years	-0.026 (-0.38)	-0.026 (-0.37)			0.010 (0.13)	0.010 (0.13)
Firm age >6 & ≤ 8 years	-0.221∗ (-1.82)	-0.221∗ (-1.87)			-0.022 (-0.17)	-0.022 (-0.17)
Firm age >8 years	-0.273∗∗ (-2.21)	-0.273∗∗ (-2.62)			0.081 (0.63)	0.081 (0.67)
Firm size (total labour force)	0.004 (0.66)	0.004 (0.56)			0.015∗ (1.80)	0.015∗ (1.70)
Profitability-unchanged	-0.164 (-1.56)	-0.164 (-1.58)			0.088 (0.73)	0.088 (0.74)
Profitability –increased	-0.301∗∗∗ (-3.11)	-0.301∗∗∗ (-3.07)			0.081 (0.73)	0.081 (0.74)
Ownership (solo = 1)	-0.147∗∗ (-2.10)	-0.147∗∗ (-2.01)			0.137∗ (1.75)	0.137∗ (1.74)
Firm formation (government = 1)	-0.167∗ (-1.75)	-0.167∗ (-1.72)			0.367∗∗∗ (3.39)	0.367∗∗∗ (3.41)
Access to BDS (yes = 1)	-0.121∗ (-1.79)	-0.121∗ (-1.77)			0.136∗ (1.79)	0.136∗ (1.79)
Education-primary	0.144 (1.29)	0.144 (1.29)			-0.072 (-0.59)	-0.072 (-0.59)
Education-secondary	0.054 (0.53)	0.054 (0.50)			0.053 (0.47)	0.053 (0.46)
Education-college+	-0.066 (-0.55)	-0.066 (-0.52)			0.374∗∗ (2.79)	0.374∗∗ (2.77)
Sex (male = 1)	0.059 (0.86)	0.059 (0.89)			-0.038 (-0.50)	-0.038 (-0.51)
Rental cost					-0.147∗∗ (-2.50)	-0.147∗∗ (-2.50)
Observations	1321				1321	

Atanhrho	-1.537∗∗∗ (-7.34)		-1.537∗∗∗ (-6.46)			

Note: Z values are provided in brackets ∗∗∗<0.01, ∗∗<0.05, ∗<0.1.

The Z-values for the estimation results with and without robust standard errors are quite similar suggesting heteroskedasticity is not a serious problem.

## Footnotes

1 For instance, since the onset of interbank borrowing activity in 1998, only 23 transactions worth about 259 million Birr (7.2 million USD at current exchange rate) have been realized. Moreover, though there is now one bank branch for every 17 thousand people, over 90% of the population (most of them in the countryside) remain excluded from banking financial services.

2 In Ethiopia, based on government definition of enterprise-size classification, micro-business have employment level of at most six workers and small enterprises have employment level between six and thirty workers.

3 Among those firms that accessed external finance within two years before the conduct of the specific survey, 81% got credit from microfinance institution, 4% from banks, and the remaining from informal sources.

4 Another specification was also estimated by controlling for the effects of 11 firm locations and 12 sub-sector categories. The results (not included in this paper) were quite similar to those reported in Table 4. Table 6 and table 8 (in the appendix) present results of multi-colinearity test and adjustment for possible heteroskedatsic effects.
